# Evaluation of hyperprolactinemia risk factors in infertile women referred to Yazd Infertility Center: A cross-sectional study

**DOI:** 10.18502/ijrm.v19i12.10059

**Published:** 2022-01-12

**Authors:** Elahe Dehghan, Nasim Namiranian, Akram Ghadiri-Anari, Seid Kazem Razavi Ratki, Reyhaneh Azizi

**Affiliations:** ^1^Shahid Sadoughi University of Medical Sciences, Yazd, Iran.; ^2^Diabetes Research Center, Shahid Sadoughi University of Medical Sciences, Yazd, Iran.

**Keywords:** Hyperprolactinemia, Infertility, PCOS.

## Abstract

**Background:**

Hyperprolactinemia is one of the most common causes of infertility in women. The prevalence of pituitary tumors is 25-30% among infertile participants with hyperprolactinemia.

**Objective:**

The aim of this study was to describe the causes of hyperprolactinemia in infertile women referred to Yazd Infertility Center.

**Materials and Methods:**

This cross-sectional study was conducted with 182 infertile women with hyperprolactinemia who were referred to Yazd Infertility Center from February 2018 to October 2019. Serum prolactin was assessed by the human prolactin ELISA kit according to the Padtan Gostar Isar protocol. The age, duration of infertility, causes of hyperprolactinemia, and type of infertility treatment were noted. The MRI findings were added.

**Results:**

The mean age of participants was 28.9 ± 0.36 yr and the prolactin level was 76 ± 8.97 ng/ml. The etiology of hyperprolactinemia among the study participants was 35 participants (19.2%) with pituitary adenoma, 47 participants (25.8%) with polycystic ovary syndrome, 14 participants (7.7%) with pituitary adenoma and polycystic ovary syndrome, and 86 participants (47.3%) with idiopathic hyperprolactinemia. The results of this study showed that there was no statistically significant difference between the mean prolactin levels in participants with different causes of hyperprolactinemia (p = 0.31).

**Conclusion:**

Idiopathic hyperprolactinemia and polycystic ovary syndrome are the most common reasons for hyperprolactinemia.

## 1. Introduction

Hyperprolactinemia (Hyper-PRL) is a common endocrine disorder (1). Hyper-PRL occurs in both genders at any age (2). The annual incidence of Hyper-PRL in men and women is 1.4 and 8.7 per 100,000 persons, respectively. The most common causes of Hyper-PRL are pregnancy, pituitary adenoma, intracranial tumors, prolactin stimulants (3), and pharmacological conditions such as the administration of estrogen or heavy metals (4). Hyper-PRL has also been observed in primary hypothyroidism, chronic renal failure, and hemodialysis (5). Moreover, some participants with Hyper-PRL are diagnosed as having idiopathic Hyper-PRL (3). If participants have a continuously raised prolactin level and other causes have been excluded, a pituitary tumor is assumed. Magnetic resonance imaging (MRI) is the most sensitive imaging technique for recognizing pituitary tumors (6, 7).

Hyper-PRL is associated with short- and long-term consequences including menstrual disorders, decreased quality of life, decreased bone mineral density, and sexual dysfunction. Hyper-PRL affects fertility by impairing gonadotropin-releasing hormone (GnRH) secretion and interfering with ovulation (8, 9). Common manifestations of Hyper-PRL are anovulation, oligomenorrhea, amenorrhea, and galactorrhea (8, 10). Morphological changes seen in the follicles in hypothyroidism can be due to the production of higher levels of prolactin which may block the secretion and function of gonadotropins (11).

Dopamine agonists are considered the first-line therapy for Hyper-PRL. Dopamine agonist therapy can restore the normal level of prolactin and gonadal action (12). Hyper-PRL is one of the most frequent etiologies of infertility in women. Almost all Hyper-PRL participants need MRI imaging during diagnostic approaches, but MRI is not easily accessible or affordable, and it is time consuming. About 25-30% of these participants have pituitary tumors (1).

The aim of the current study was to evaluate the causes of Hyper-PRL in infertile women referred to the Yazd Infertility Center.

## 2. Materials and Methods

This cross-sectional study was conducted with 182 Hyper-PRL infertile women who were referred to Yazd Infertility Center from February 2018 to October 2019.

The inclusion criteria were: married women with prolactin higher than 20 ng/ml; and normal range of thyrotropin stimulating hormone (0.4 to 4.0 mIu/lit). The exclusion criteria were: no cirrhosis or kidney failure; and no history of medication which increases prolactin levels such as hormonal or antipsychotic drugs. Information such as age, duration of infertility, reasons for Hyper-PRL, MRI findings and type of infertility treatment were extracted from participants' medical records. After 8-12 hr of night fasting, a blood sample was collected from all participants using a 5 ml disposable syringe and their serum was separated by centrifugation (Eppendorf). Serum prolactin was assessed using the human prolactin ELISA kit according to the Padtan Gostar Isar protocol.

### Ethical considerations

This study was approved by the Ethical Committee of Shahid Sadoughi University (Code: IR.SSU.MEDICINE.REC.1397.227).

### Statistical analysis

Data were demonstrated by the mean, standard deviation (SD), frequency, and percentages. Data were analyzed using Chi-square test, *t* test, and ANOVA. All analyses were performed in the Statistical Package for the Social Sciences (SPSS) software version 22.0 (SPSS Inc., Chicago, USA). P-values less than 0.05 were considered significant.

## 3. Results

This study was conducted with 182 infertile women with Hyper-PRL. The mean ± SD age of participants was 28.9 ± 0.36 yr (age range of 20-42 yr). The mean prolactin level, age, and duration of infertility are presented in table I. The frequency of Hyper-PRL causes is presented in table II. As demonstrated in table II, idiopathic Hyper-PRL was the most common reason for Hyper-PRL (47.3%). Also, evaluation of the mean level of prolactin in terms of the causes of Hyper-PRL showed that there was no relationship between the level of prolactin and the causes of Hyper-PRL (p = 0.31).

There was no significant difference in the mean duration of infertility in participants with different causes of Hyper-PRL (p = 0.15). In addition, no significant difference was observed between the mean age of participants across the different causes of Hyper-PRL (p = 0.47); however, the mean age of participants in the pituitary adenoma group (29.36 ± 5.25) was significantly greater than in the polycystic ovary syndrome (PCOS) and adenoma group (26.7 ± 3.65) (p = 0.01). The frequency distribution of infertile women in terms of treatment types is shown in table III. The largest proportion of participants (47.8%) underwent in vitro fertilization therapy. Table IV shows the frequency of different causes of Hyper-PRL; no relationship was observed between the frequency of different causes of Hyper-PRL and the type of treatment (p = 0.33).

**Table 1:** The mean prolactin level, age, and duration of infertility


**Variables**	**Mean ± SD**	**Minimum**	**Maximum**
**Prolactin (ng/ml)**	76.00 ± 8.97	19.55	850.20
**Age (yr)**	28.90 ± 0.36	20	42
**Duration of infertility (yr)**	5.04 ± 0.29	1	25

**Table 2:** The frequency distribution of causes of Hyper-PRL


**Causes**	**N (%)**
**Idiopathic Hyper-PRL**	86 (47.3%)
**PCOS**	47 (25.8%)
**Pituitary adenoma**	35 (19.2%)
**PCOS + pituitary adenoma**	14 (7.7%)
**Total**	182 (100%)
PCOS: Polycystic ovary syndrome, PRL: Prolactin	

**Table 3:** The frequency distribution of infertile women in terms of treatment types


**Type of infertility treatment**	**N (%)**
**IVF**	87 (47.8%)
**Medication treatment**	69 (37.9%)
**IUI**	16 (8.8%)
**Surgery**	5 (2.7%)
**Reception of donated ovule**	2 (1.1%)
**Fetal donation**	2 (1.1%)
**Missed**	1 (0.5%)
**Total**	182 (100%)
IVF: In vitro fertilization, IUI: Intrauterine insemination	

**Table 4:** The frequency of different causes of Hyper-PRL, in relation to the type of treatment


[1.455in,lr]**Cause of hyperprolactinemia** **Type of treatment**	**IVF**	**IUI**	**Medication treatment**	** Other methods**	**p-value**
**Idiopathic**	44 (51.2)	6 (70.0)	31 (36.0)	5 (5.8)	0.33
**PCOS**	23 (48.9)	4 (8.5)	17 (36.2)	3 (6.4)	
**Pituitary adenoma**	18 (51.4)	4 (11.4)	13 (37.1)	0 (0)	
**Both (PCOS + pituitary adenoma)**	2 (14.3)	2 (14.3)	8 (57.1)	2 (14.3)	
**Total**	87 (47.8)	16 (8.8)	69 (37.9)	10 (5.5)	
Values reported as n (%). P-value was obtained from Chi-square test. PCOS: Polycystic ovary syndrome, IVF: In vitro fertilization, IUI: Intrauterine insemination					

## 4. Discussion

The mean duration of infertility in the present study was 5.04 ± 0.29 yr. One study of infertile women with Hyper-PRL reported that the mean duration of infertility was 9.1 yr (13). Another study performed on 95 infertile women showed that the mean duration of infertility in these participants was 8.9 yr (14). It seems that the shorter duration of infertility found in the present study may have been due to the referral of younger participants to Yazd Infertility Center during 2019.

Hyper-PRL may lead to defective ovulation and decreased fertility (15). Several possible reasons for Hyper-PRL have been reported in studies. Stimulants such as thyrotropin-releasing hormone, vasoactive intestinal peptide, estrogen, and dopamine receptor antagonists induce prolactin secretion (16). Also, studies have shown that excessive secretion of prolactin reduces the pulsatile release of GnRH and impairs normal gonadal steroid secretion, which leads to positive feedback effects at the pituitary and hypothalamic levels, and ultimately infertility (15).

In the present study, idiopathic Hyper-PRL was the most common reason for Hyper-PRL with a frequency of 47.3% in infertile participants. The results of a number of studies are in line with these findings. One study demonstrated that idiopathic Hyper-PRL was seen in 8.5%-40% of participants with Hyper-PRL (16). In another study of 183 women with hyper-PRL, most participants had idiopathic Hyper-PRL. The high frequency of idiopathic Hyper-PRL found in our and these studies could be due to not re-examining the prolactin test or testing after the menstrual phase, or long-term use of drugs that interfere with dopamine (a prolactin secretion inhibitor), even if some time has passed since the drug was stopped. Another possibility may be the consumption of drugs that the patient did not mention in the history or that were not on the list of drugs that cause Hyper-PRL but increased prolactin (15). Other studies have reported that idiopathic Hyper-PRL may be due to genetics, macroprolactinemia, or prolonged use of drugs that interfere with dopamine (15, 17).

In these participants, fertility may increase via long-term use of dopaminergic drugs. This treatment prompts ovulatory cycles and normalizes the defective luteal phase. Dopaminergic therapy should be continued for at least one yr since more than 50% of pregnancies occur six months after medication. Stimulation of the ovary with pulsatile administration of GnRH and gonadotropin may lead to ovulatory cycles and fertility in the infertile participants (18).

In addition, while PCOS is often considered the most common cause of Hyper-PRL in infertile women, in the present study it was ranked as second. In a study aimed at assessing PRL levels in women with PCOS, Hyper-PRL was observed (19). Hyper-PRL in participants with PCOS may reflect a deficiency of dopamine (20). One of the possible mechanisms for hyperprolactinemia in women with PCOS-induced secretion of luteinizing hormone is accelerating GnRH pulsatility and decreasing dopaminergic tone (21). PCOS and Hyper-PRL have been reported to be the most common etiologies of anovulation in females (21). Various hypotheses have been proposed for this. Several studies have shown that in women with PCOS, the secretion of prolactin is increased under the action of estrogen. However, a number of studies have shown that the use of oral contraceptive medications such as estrogen does not lead to an increase in prolactinoma size (22). Hyper-PRL may be associated with insulin resistance and glucose intolerance in participants with PCOS. It has been shown that prolactin can be associated with enhanced concentration of fatty acids, downregulation of insulin receptors, and defects in insulin function (23). One study confirmed insulin resistance in Hyper-PRL subjects and showed the role of prolactin in mediating insulin resistance (24). Thus, if a high percentage of Hyper-PRL cases are due to PCOS, a course of treatment for this disease and prolactin re-assessment is necessary.

Pituitary adenoma accounted for 19.2% of Hyper-PRL cases. Some studies have shown that Hyper-PRL may be mainly due to pituitary tumors (25). Other studies have reported that the cause of Hyper-PRL is mostly due to pituitary adenomas (26-28). A study on 87 infertile women aged 31-40 yr with Hyper-PRL showed that 75.8% of participants suffered from microadenoma. Moreover, participants with pituitary adenoma had a significantly higher level of prolactin than others, which is not consistent with our study. The reason for this difference may be the time of PRL check. In that study, participants with PCOS and pituitary adenoma were younger than participants with pituitary adenoma, which is consistent with our study (25). Differences in findings may be due to the MRI being carried out after repeated measurement of serum prolactin.

The high percentage of idiopathic Hyper-PRL may be due to macroprolactinemia, which is not routinely checked for in Iranian laboratories. Prolactin should be measured in the follicular phase and re-assessed, and if it is elevated then MRI study is recommended.

## 5. Conclusion

Idiopathic Hyper-PRL and PCOS are the most common reasons for Hyper-PRL. Pituitary adenoma appears to be more common in younger PCOS participants, but further studies are needed to confirm this.

##  Conflict of Interest

The authors declare that there is no conflict of interest.
